# Characteristics and Function of Sulfur Dioxygenase in Echiuran Worm *Urechis unicinctus*

**DOI:** 10.1371/journal.pone.0081885

**Published:** 2013-12-02

**Authors:** Litao Zhang, Xiaolong Liu, Jianguo Liu, Zhifeng Zhang

**Affiliations:** Key Laboratory of Marine Genetics and Breeding, Ministry of Education, Ocean University of China, Qingdao, China; Universidade Nova de Lisboa, Portugal

## Abstract

**Background:**

Sulfide is a common toxin to animals and is abundant in coastal and aquatic sediments. Sulfur dioxygenase (SDO) is thought to be the key enzyme involved in sulfide oxidation in some organisms. The echiuran worm, *Urechis unicinctus*, inhabits coastal sediment and tolerates high concentrations of sulfide. The SDO is presumably important for sulfide tolerance in *U. unicinctus*.

**Results:**

The full-length cDNA of *SDO* from the echiuran worm *U. unicinctus*, proven to be located in the mitochondria, was cloned and the analysis of its sequence suggests that it belongs to the metallo-β-lactamase superfamily. The enzyme was produced using an *E. coli* expression system and the measured activity is approximately 0.80 U mg protein^−1^. Furthermore, the expression of four sub-segments of the *U. unicinctus SDO* was accomplished leading to preliminary identification of functional domains of the enzyme. The identification of the conserved metal I (H113, H115, H169 and D188), metal II (D117, H118, H169 and H229) as well as the potential glutathione (GSH) (R197, Y231, M279 and I283) binding sites was determined by enzyme activity and GSH affinity measurements. The key residues responsible for SDO activity were identified by analysis of simultaneous mutations of residues D117 and H118 located close to the metal II binding site.

**Conclusion:**

The recombinant SDO from *U. unicinctus* was produced, purified and characterized. The metal binding sites in the SDO were identified and Y231 recognized as the mostly important amino acid residue for GSH binding. Our results show that SDO is located in the mitochondria where it plays an important role in sulfide detoxification of *U. unicinctus*.

## Introduction

Sulfide, a common toxin, may be harmful for organisms by reducing the affinity of hemoglobin to oxygen [1], inhibiting the activity of cytochrome c oxidase and succinate oxidase complexes [2], [3], depolarizing mitochondria [4], inducing apoptosis [5], and causing oxidative damage to RNA and DNA [6]. In marine sediments sulfide accumulates because of the existence of anaerobic sulfate-reducing bacteria [7]. Animals in permanent burrows are frequently exposed to sulfide during low tides; for example, sulfide could reach 66 µM in the burrow water where the echiuran worm *Urechis caupo* lives [8] and variety of defensive responses are adopted by animals living in sediments. Mitochondrial oxidation is considered the primary pathway used to detoxify sulfide in the worms living in sediments where prolonged exposure to toxic sulfide occurs [9].

**Figure 1:**
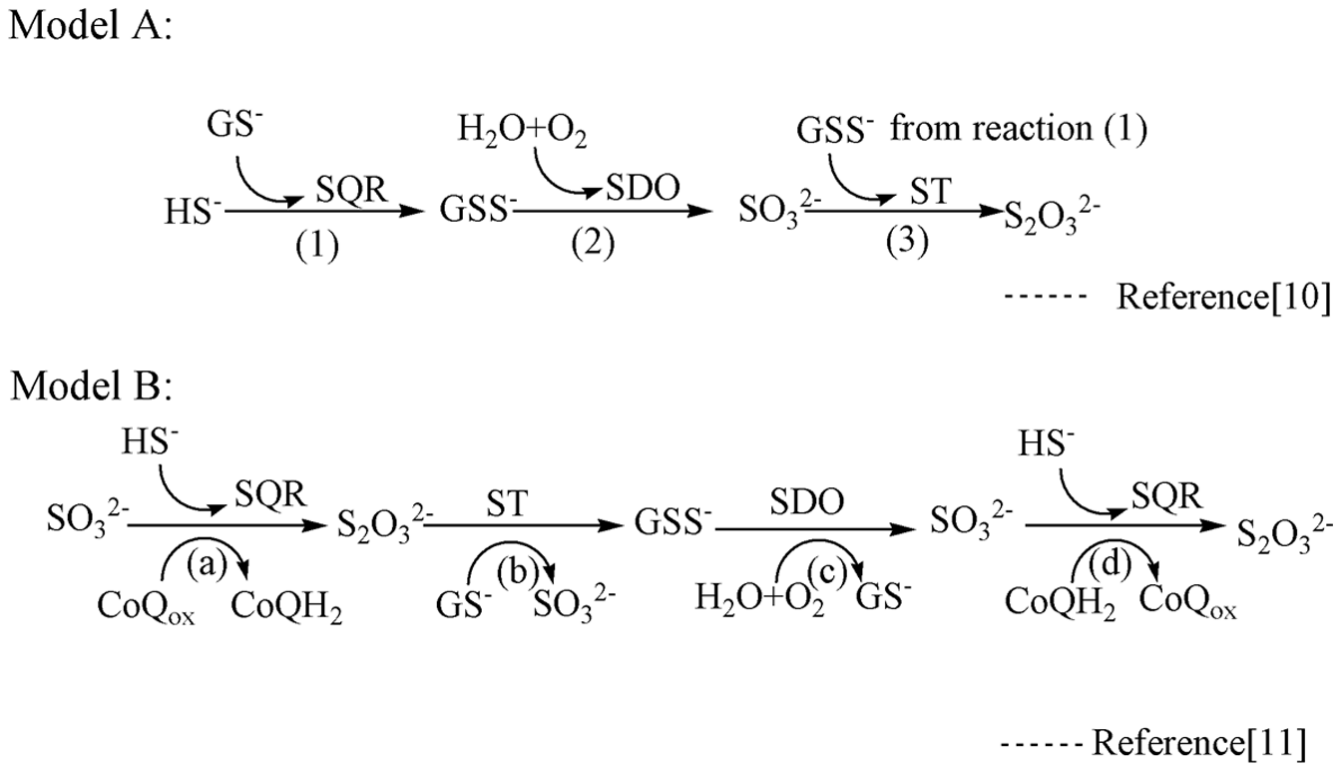
The two proposed sulfide oxidation models. Abbreviations: SQR, sulfide: quinine oxidoreductase; ST, sulfur transferase; SDO, sulfur dioxygenase; GS^−^, glutathione; GSS^−^, glutathione persulfide. Model A: The sulfide can be converted to GSS^−^ by SQR, and the sulfane sulfur of GSS^−^ is oxidized to sulfite using O_2_ by SDO in the mitochondrial-matrix; finally, the generated sulfite is converted by ST catalysis to thiosulfate, which is less toxic to the organism. Model B: Thiosulfate biosynthesis occurs in the first step of sulfide oxidation catalyzed by SQR with sulfite as the acceptor of the sulfane sulfur, then thiosulfate can act as the ST substrate to produce GSS^−^ and regenerate the sulfite; finally, the sulfane sulfur of GSS^−^ is oxidized to sulfite using O_2_ by SDO. The newly generated sulfite could then enter the cycle again.

In mitochondria, an enzymatic system involving three enzymes, sulfide: quinine oxidoreductase (SQR), sulfur dioxygenase (SDO) and sulfur transferase (ST), are involved in oxidative sulfide detoxification. Two models for sulfide oxidation [10], [11] have been proposed as shown in Figure 1. SDO plays an essential role in both by oxidizing sulfane sulfur of glutathione persulfide (GSS^−^) to sulfite by using O_2_. SDO in humans was initially recognized as ETHE1 protein (ethylmalonic encephalopathy 1) since it was recognized that *ETHE1* gene mutation leads to ethylmalonic encephalopathy (EE) [12]. Recently, Tiranti et al. [13] suggested that ETHE1 possesses SDO activity and is involved in the oxidation of sulfide since SDO activity 1) is absent in EE patients and *ETHE1* ^−/−^ mice, and 2) increases when human ETHE1 is overexpressed in Hela or *E. coli* cells. Moreover, in *Arabidopsis thaliana*, ETHE1 also catalyzes the GSS^−^-dependent activity with consumption of oxygen at a rate of 7.95±0.71 µmol O_2_ min^−1^ mg^−1^ [14]. Thus, at present, the function of ETHE1 is mostly focused in its SDO activity and the biochemical characterization and kinetic properties of the human enzyme shows a Michaelis constant (K_M_) for GSSH of 0.34±0.03 mM and a V_max_ of 113±4 µmol min ^−1^ mg protein ^−1^ [15]. To date, most of the research concerning the SDO enzyme has been restricted to mammal and plant sources and no information in invertebrates, in particular those that have sulfide tolerance, was reported.

The echiuran worm *Urechis unicinctus* is mainly distributed in China, Korea, Russia and Japan, and inhabits marine sediments, especially intertidal and subtidal mudflats [16], [17]. It has been reported that *U. unicinctus* can tolerate, use and metabolize environmental sulfide [18], [19], [20]. Furthermore, the presence of the *U. unicinctus SQR* was revealed in different tissues and upon exposure to different sulfide concentrations at the mRNA, protein and enzyme activity levels [21], [22]. This study aims at increasing our understanding of sulfide metabolic adaptation as well as exploring the function and catalytic mechanism of SDO in *U. unicinctus*. The full length *SDO* cDNA as well as four sub-segmental sequences were cloned and expressed in *E. coli* allowing for the elucidation of domains responsible for enzyme activity and the catalytic mechanism of *U. unicinctus* SDO.

## Materials and Methods

### Cloning of target full length cDNA in *U. unicinctus*

The nested degenerate primers for cloning *SDO* cDNA fragment were designed according to the evolutionary conserved domains of *SDO* cDNA in other species obtained from the National Center for Biotechnology Information (NCBI). The primary PCR was conducted using the forward (5′-CAYGCNGAYCAYATHACNGG-3′) and reverse degenerate primers (5′-GTARTCRTGNGCNGGRTANA-3′), and the body wall cDNA of *U. unicinctus* as a template. Semi-nested PCR was conducted using 2000× diluted primary PCR product as the template and the semi-nested reverse degenerate primer was changed to 5′-TGGAARTCNGTNCKNCCRCA-3′. The PCR product was purified, subcloned into pMD18-T vector (Takara, Otsu, Japan), and then transformed into *E. coli*-DH5α competent cells (Takara). The obtained fragments were sequenced using an ABI PRISM 3730 DNA sequencer (Applied Biosystems, Foster City, CA, USA) and the sequence alignment was performed using Blastx from the NCBI, and was preliminarily confirmed as the *SDO* sequence fragment.

The 3′ and 5′ RACE ready first-strand cDNA was synthesized using the SMARTer™ RACE kit according to the manufacturer's instructions (Clontech, Mountain View, CA, USA). The specific primers, GSP-5′ (5′-GCCATCCCTTTCTCATGCCAAACAT-3′) and GSP-3′ (5′-TGTGATTGCCGAATGCAGTCAAGCT-3′), were designed to clone the 5′ and 3′ regions of *SDO*, respectively. The RACE PCR reactions were performed using the Advantage II Polymerase Mix (Clontech). The 3′ and 5′-RACE products were gel-purified, subcloned, sequenced, and assembled with SeqMan Pro (DNA STAR, Madison, WI, USA).

### Sequence analysis

The sequence similarity of SDO with those from other species was analyzed using BLAST (http://blast.ncbi.nlm.nih.gov/Blast.cgi), and the protein structure and biochemical properties were predicted using the ExPASy proteomics server (http://www.expasy.org/tools/). The three-dimensional sequence model was predicted with the alignment mode in Swiss Model using the SDO protein from *A. thaliana* (PDB ID: 2GCU) as the template [23], [24], [25]. The multiple alignments of SDO were generated with ClustalX 2.1 and the phylogenetic tree was constructed using the MEGA program 5.0 by the Neighbor-Joining method using the Poisson correction amino acid substitution model and the complete deletion gaps option. Bootstrap values from 1000 replicates were calculated and indicated at branch points on the neighbor-joining tree.

### Prokaryotic expression, purification and refolding of *U. unicinctus* SDO

**Figure 2:**
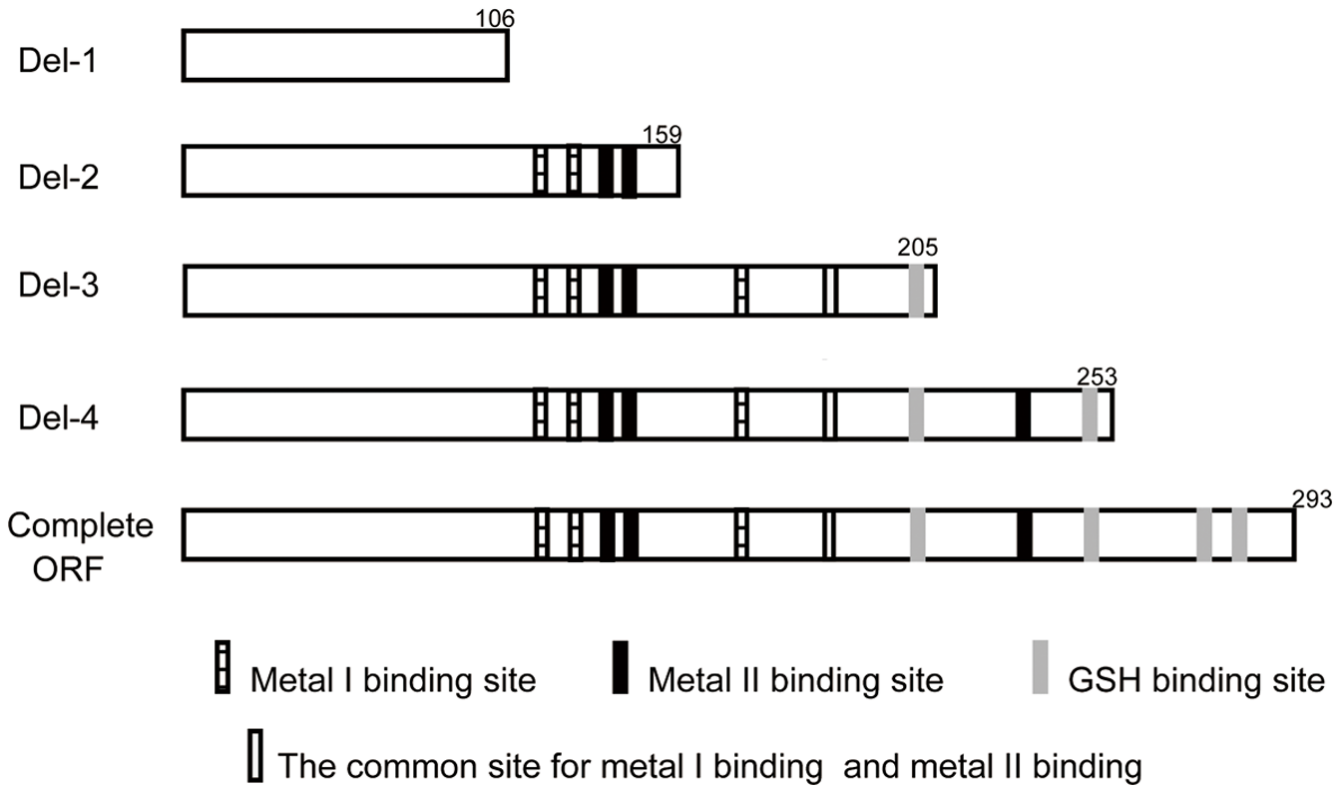
Schematic diagram of SDO sub-segment expression. Del-1: no structural amino acid site; Del-2: two metal I binding site residues, two metal II binding site residues and no GSH binding site; Del-3: complete metal I binding site, three metal II binding site residues and one GSH binding site; Del-4: complete metal I binding site and metal II binding site as well as three GSH binding sites; complete ORF: complete metal I binding sites and metal II binding sites as well as GSH binding sites. The numbers indicated the expressed sub-segment amino acid length.

Based in protein structural predictions, four sub-segments (Del-1-Del-4) of the *U. unicinctus SDO* open reading frame (ORF) and the complete ORF sequence were chosen, as shown in Figure 2, and amplified using the primers in Table 1. The obtained cDNA sequences were cloned into the pET28a plasmid, sequenced and transformed into *E. coli* BL21 (DE3). These expressed proteins contained 6-His tags at both C-terminal and N-terminal for purification purposes on a nickel affinity column. An overnight culture of *E. coli* BL21 (DE3) was grown in 1 mL Luria Bertani (LB) with kanamycin (30 µg/mL) at 37°C and then inoculated into 100 mL of LB media also supplemented with kanamycin. When the OD_600_ reached 0.5 expression was induced by adding 1 mM IPTG (isopropyl- β-D-1-thiogalactopyranoside) and the culture was grown for an additional 5 h period. The cells were then harvested by centrifugation at 10,000 *g* for 10 min at 4°C, suspended in 50 mM phosphate buffered solution (PBS, pH 7.4), and broken by ultrasonication on ice. The target recombinant proteins were found primarily in inclusion bodies. The five proteins were dissolved in 4 mL of buffer composed of 8 M urea, 10 mM Tris-HCl (pH 8.0) and 0.1 M trisodium phosphate. The recombinant proteins were purified by Ni-NTA affinity chromatography according to the manufacturer's protocol (Novagen, Darmstadt, Germany), and the purity of the eluted samples was analyzed by SDS-PAGE. The recombinant proteins were refolded by adding the refolding buffer (50 mM glycine, 100 mM phosphate buffer, 200 mM NaCl, 5 mM EDTA, 5 mM FeCl_2_, 10 mM DTT) with stirring to dilute the 8 M urea to 1 M, and then allowing the solution to stir at 4°C for a further 12 h. The urea was removed by dialysis; the soluble and aggregated fractions of the renaturation mixture were separated by centrifugation (10,000 *g*, 4°C, and 10 min) and analyzed by SDS-PAGE to confirm that the soluble fraction was successfully refolded.

**Table 1:** Primers for the five cDNA sequences of *U. unicinctus SDO.*

	Forward primer	Reserved primer	Size (bp)
Del-1	GAGCTCATGCTTTCGTCCGTATGTGG	CTCGAGTAGATTGAGCCCGAGTTCTTT	318
Del-2	GAGCTCATGCTTTCGTCCGTATGTGG	CTCGAGGAATTGACCAAACTCGATACC	477
Del-3	GAGCTCATGCTTTCGTCCGTATGTGG	CTCGAGTGAGCTTCCTTGTTGGAAATC	615
Del-4	GAGCTCATGCTTTCGTCCGTATGTGG	CTCGAGAATTGGTTTAGTGAGGCGGG	759
Complete ORF	GAGCTCATGCTTTCGTCCGTATGTGG	CTCGAGGGACTTGCTTGGGGGTGATT	879

### Double mutant of *U. unicinctus* SDO

Double mutagenesis was conducted using a fast mutagenesis system kit (Transgen, Beijing, China) and the mutations were generated using the primers, forward: ACACACATGTGCACGCTGA***GGC***TGTGACTGGTA and reverse: ***GCC*** TCAGCGTGCACATGTGTGTTGACTGCATA (the bold and italic letters show the bases deviating from the original sequence) to obtain the double mutant, D117E/H118A. The resulting sequence was analyzed by DNA sequencing and the refolded mutated SDO was attained as described above.

### Enzyme activity assays

SDO activity (1U =  1 µmol GSS^−^ min^−1^) was measured by the consumption of the substrate GSS^−^ according to the method of Hildebrandt and Grieshaber [10]. The reaction mixture (2 mL) consisted of 0.1 M potassium phosphate buffer (pH 7.4), 1 mM GSH and 3 µg L^−1^ of the refolded SDOs. The reaction was started by adding 30 µL of a saturated acetonic sulfur solution. Samples and 250 µL aliquots were taken from the reaction mixture at various time intervals and incubated with 375 µL HCl (10 M) and 375 µL methylene blue (75 µM) at room temperature for 30 min. The absorption of oxidized methylene blue was measured at 670 nm, and subtracted from that of the blanks containing buffer instead of the sample. GSS^−^ consumption was determined considering the amount of methylene blue reduced.

### Enzyme kinetics analysis

To explore the kinetic characterization of the recombinant SDOs, the substrate GSS^−^ was prepared by anaerobically mixing GSH (10 mM in 200 mM sodium phosphate, pH 7.4) with saturated acetonic sulfur solution. The GSS^−^ concentration was determined as described above. The reaction mixture (2 ml) contained 0.1 M potassium phosphate buffer (pH 7.4), GSS^−^ (gradient concentrations) and 3 µg L^−1^ of the refolded SDOs. The reactions were started by adding enzyme SDO preparations, and oxygen consumption was recorded using the Oxytherm liquid-phase oxygen measurement system (Hansatech, Pentney, U.K.) at 25°C. Kinetic parameters were calculated by a non-linear least square analysis of the data fitted to the Michaelis–Menten equation using the enzyme kinetics module of Sigmaplot version 12.0 (Systat Software, Erkrath, Germany).

### GSH-affinity determination

A solution containing 3 µg L^−1^ refolded SDO in 100 mM phosphate buffer in the presence of 50 µM GSH was incubated for 30 min and then subjected to ultrafiltration (10,000 *g*, 4°C, and 30 min) using a centrifugal filter device (Amicon Ultra-4 10K, Millipore, Billerica, MA, USA), which allowed the GSH, but not the GSH bound to the SDO, to pass through the filter. The filtrate was analyzed for GSH using a total glutathione assay kit (Jiancheng, Nangjing, China). The GSH binding ability was calculated by determining the loss of GSH in the filtrate.

### Western blot analysis

Mitochondria were isolated from the midgut of *U. unicincuts* according to the methods described by Schroff and Schöttler [26] with slight modifications. The midgut tissue and mitochondrial total protein were extracted using the tissue protein extraction kit (Cwbio, Beijing, China). SDS-PAGE and western blotting were carried out as described [21]. A polyclonal antibody of *U. unicinctus* SDO was prepared by injecting purified recombinant SDO into New Zealand white rabbits at a titer of 1∶ 25,600.

### Statistical analysis

All data are presented as mean±SE. Significant differences among means were tested by one-way analysis of variance (ANOVA) followed by Duncan's multiple comparison procedure using the SPSS statistical package version 18.0 (IBM SPSS, Chicago, IL, US) at a significance level of p<0.05.

## Results

### Sequence characteristics of *U. unicinctus* SDO

A cDNA fragment of 260 bp was obtained by RT-PCR using degenerate primers, and two fragments of 886 bp and 1240 bp were cloned by 5′ and 3′ RACE, respectively, and then assembled to a 1976-bp full-length *SDO* cDNA (GenBank accession number: HQ730921.1).

**Figure 3:**
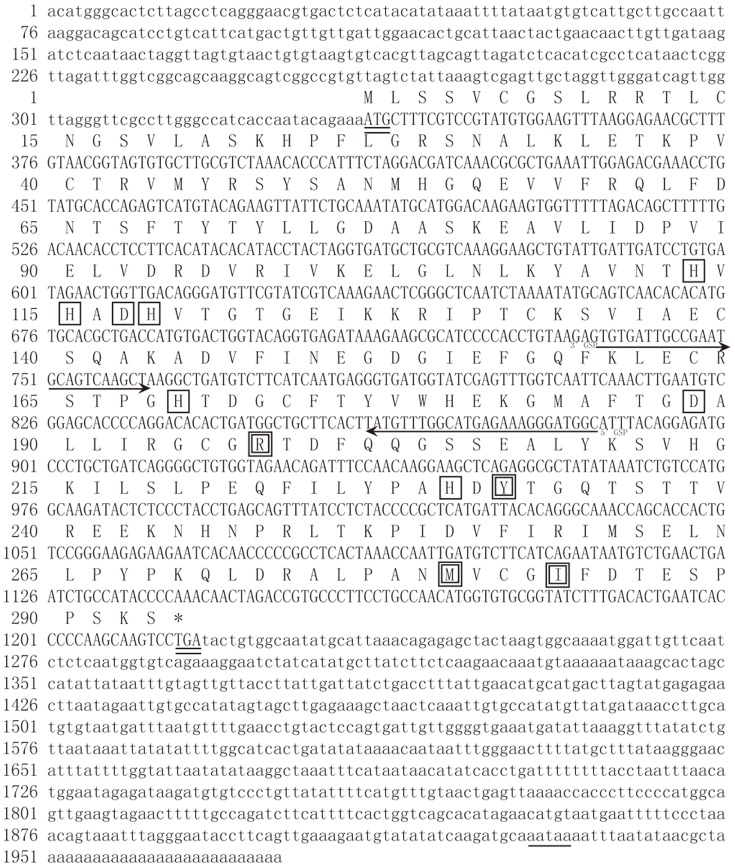
Nucleotide sequence and predicted amino acid sequence of *SDO* in *U. unicinctus.* Start (ATG) and stop (TGA) codons, double lines; RACE gene specific primers (GSP), indicated by an arrow; the AATAAA polyadenylation signal is underlined; Conserved metal binding sites are boxed in black; Conserved GSH binding sites are double boxed.

**Figure 4:**
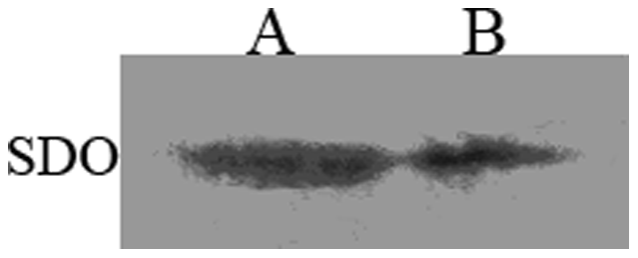
Mitochondrial location of SDO in *U. unicinctus.* A. Midgut total protein; B. Midgut mitochondrial protein.

The full length cDNA sequence of *SDO* as well as the resultant amino acid sequence is shown in Figure 3. The ORF has 882 bp, encoding a 293 amino acids polypeptide with a theoretical pI of 8.03 and molecular mass of 32.61 kDa. The conserved metal I (H113, H115, H169 and D188), metal II (D117, H118, H169 and H229) and potential GSH (R197, Y231, M279 and I283) binding sites are indicated in the amino acid sequence. The western blot analysis shows that the SDO protein is present in the mitochondria (Figure 4).

**Figure 5:**
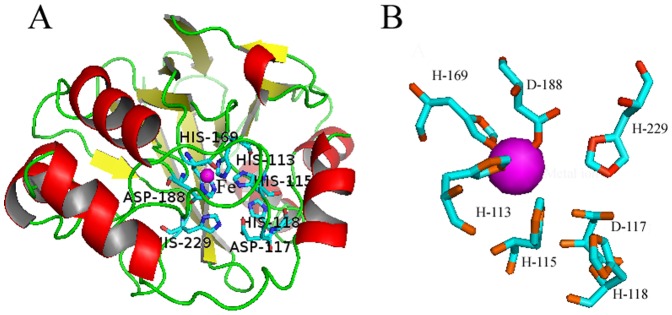
The predicted three-dimensional models of *U. unicinctus* SDO based on SDO protein in *A. thaliana* (PDB ID: 2GCU). A. The β-strands, α-helices, and loop regions are shown as yellow, red, and green ribbons, respectively. The typical β-lactamase fold and metal binding sites are labeled. B. The iron (magenta sphere) binding amino acids, H113, H169 and D188, in metal binding site I form the 2His:1Asp facial triad, the remaining residues shown are found in the metal binding site II around the iron.

According to the three-dimensional model of the *U. unicinctus* SDO protein (Figure 5), the functional domain is characterized by a typical β-lactamase fold where the metal binding sites locate, a four-layered β-sandwich fold with two mixed β-sheets flanked by α-helices. It is well known that five histidines and two aspartate residues are important for metal binding in the metallo-β-lactamase superfamily [27], [28] and these residues are also conserved in *U. unicinctus* SDO (Figure 5A). The amino acids presumably involved in the metal I binding site are the conserved H113, H169, and D188 (Figure 5B); no metal ion is predicted to be bound to the conserved metal II binding site [31].

**Figure 6:**
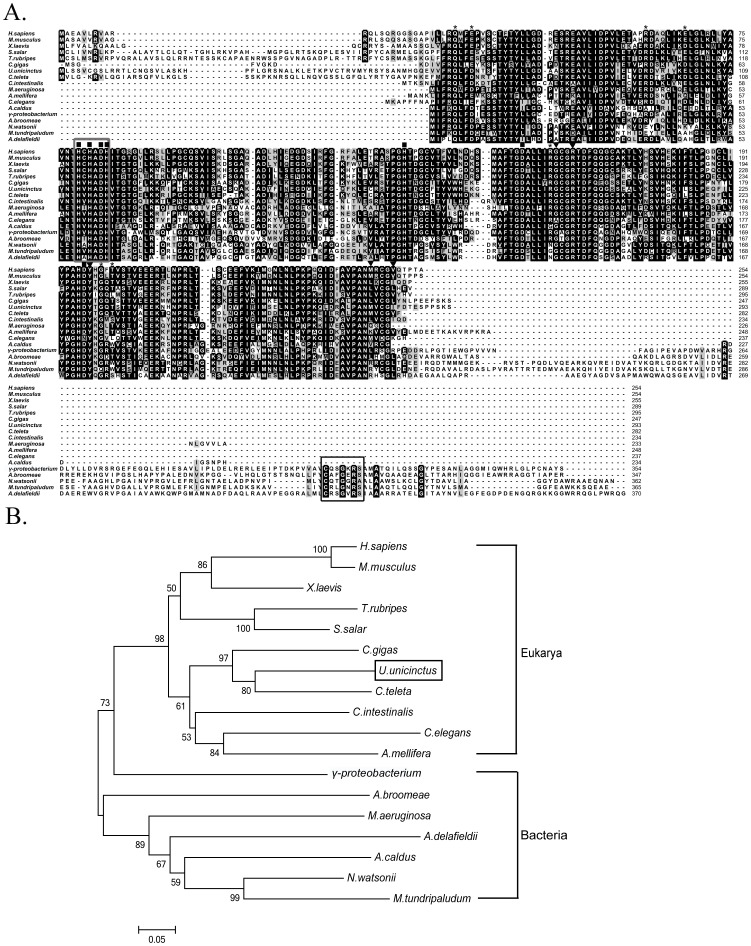
Multiple sequence alignment (A) and phylogenetic relationships (B) among the SDO sequences from different species. Identical and similar residues are highlighted in black and gray, respectively. Conserved relevant metal binding sites, GSH binding sites and dimer formation residues are indicated by ▪, ▾ and *, respectively. The β-lactamase signature motif and rhodanese active-site loop are marked with a black box and grey box, respectively. GenBank accession numbers: *Afipia broomeae* (ZP_11429388.1), *Acidithiobacillus caldus* (YP_004749948.1), *Acidovorax delafieldii* (ZP_04761469.1), *Apis mellifera* (XP_393510.1), *Caenorhabditis elegans* (NP_501684.1), *Capitella teleta* (JGI Genome), *Ciona intestinalis* (XP_002128021.1), *Crassostrea gigas* (EKC28487.1), *Homo sapiens* (NP_055112.2), *Methylobacter tundripaludum* (ZP_08782165.1), *Microcystis aeruginosa* (ZP_18834377.1), *Mus musculus* (NP_075643.1), *Nitrosococcus watsonii* (YP_003760989.1), *γ-proteobacterium HTCC2148* (ZP_05095460), *Salmo salar* (ACI68458.1), *Takifugu rubripes* (XP_003977175.1), *Urechis unicinctus* (AEV92813.1), *Xenopus laevis* (NP_001079404.1).

The results from homology analysis using Blastp show that *U. unicinctus* SDO shares some identity to SDOs from the oyster *Crassostrea gigas* (69%), the toad *Xenopus laevis* (63%), the sea squirt *Ciona testinalis* (62%), the Atlantic salmon *Salmo salar* (59%), and the mouse *Mus musculus* (58%). A ClustalX2 alignment was used to determine the overall gene conservation (Figure 6A) and indicates that SDOs among different organisms are highly conserved, especially in terms of their metal ion and GSH binding sites and also in the residues involved in the dimer formation. The phylogenetic tree constructed using the MEGA5 program based on the protein alignment (Figure 6B) shows that *U. unicinctus* SDO is most closely related to the polychaete *Capitella teleta* and the relationships displayed in the phylogenetic tree are generally in accordance with classical taxonomy.

### Identification of *U. unicinctus* SDO functional domains

**Figure 7:**
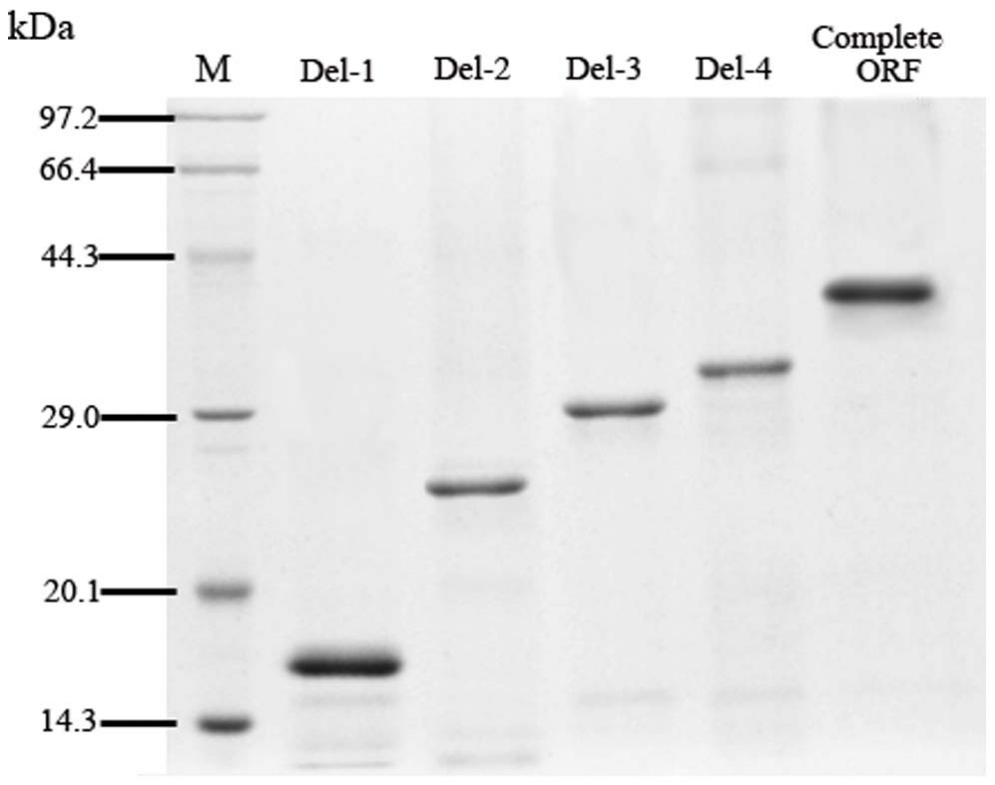
Purified recombinant SDO proteins detected by SDS-PAGE.

**Figure 8:**
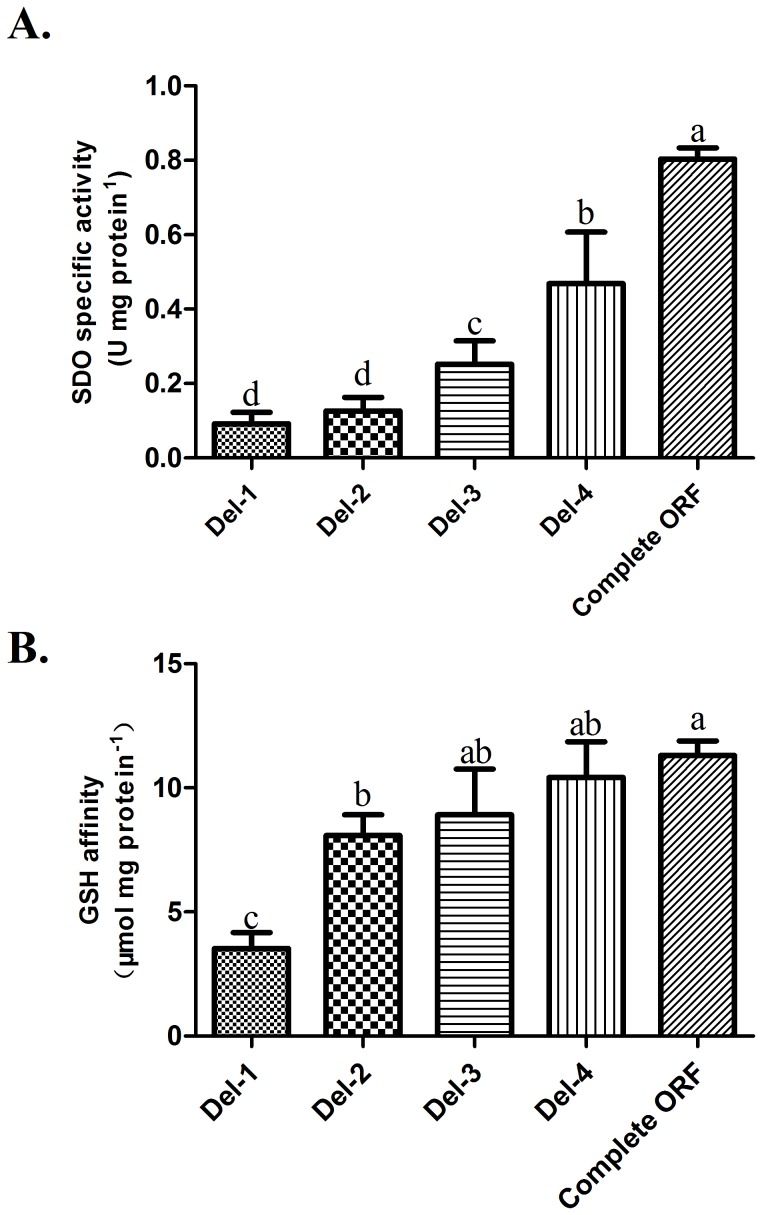
Characteristics of the five sub-segment-expressed recombinant SDO proteins. A. SDO specific activities (mean±SE, n = 3) of five recombinant SDO proteins. B. GSH affinities (mean±SE, n = 3) of the recombinant SDO proteins. Groups containing the same letters on the bar indicate no significant difference while different letters on the bar indicate a significant difference (p<0.05).

The five sequences of *U. unicinctus* SDO were expressed successfully in *E. coli* using a pET28a expression system. The recombinant proteins containing His-tags at both the C-terminal and N-terminal, were purified to 95% purity and their sizes are consistent with what was predicted (Figure 7). The SDO activities of the recombinant proteins were determined by methylene blue reduction (Figure 8A). The specific activity of the complete SDO protein is approximately 0.80 U mg protein^−1^. However, the specific activities of the truncated SDO proteins are lower than wild type enzyme. In Del-4, the SDO specific activity is significantly decreased (p<0.05) by 41.7% as compared with the wild type protein. No significant difference (p>0.05) was observed between the specific activity of Del-1 (0.09 U mg protein^−1^) and Del-2 (0.12 U mg protein^−1^), however, they were both significantly lower (p<0.05) than those measured for the rest of the enzymes.

To further understand the characteristics of truncated forms of SDO the kinetic parameters to monitor the rate of oxygen consumption during the conversion of GSS^−^ to sulfite were determined using a Clark oxygen electrode (Table 2). The V_max_ is 1.74±0.08 µmol min^−1^ mg protein^−1^ and the K_M_ is 82.5±9.9 µM for the recombinant wild type enzyme. The Del-4 protein shows a 29.3% decrease in V_max_ while the K_M_ for GSSH is unaffected. For the Del-3 protein, the V_max_ decreased to 56% and for both the Del-1 and Del-2 proteins, the V_max_ are about 20% that of the obtained for the wild type form. The values of K_M_ are not significantly different (*p*>0.05) among Del-2, Del-3, Del-4 and wild type enzyme but suffer a steeply increase up to 218.9±33.5 in the Del-1 which represents a two-fold lower affinity to the substrate than the wild type enzyme.

**Table 2:** Comparison of the kinetic properties of five recombinant SDO proteins.

	V_max_ (μmol min^−1^ mg protein^−1^)	K_M_ (μM)	k_cat_ (s^−1^)	k_cat_/K_M_ (μM^−1^ s^−1^)
Del-1	0.33±0.014^d^	218.9±33.5^A^	0.09	42.68
Del-2	0.42±0.024^d^	137.0±17.2^B^	0.16	116.13
Del-3	0.98±0.065^c^	104.6±16.7^B^	0.45	433.72
Del-4	1.23±0.063^b^	83.0±10.9^B^	0.68	822.00
Complete ORF	1.74±0.081^a^	82.5±9.9^B^	1.09	1327.22

The values with different superscripts in the same column are significantly different (p<0.05).

The GSH-affinity of the recombinant complete protein is 11.3 µmol mg protein^−1^ (Figure 8B). The GSH-affinity for Del-1 is the lowest among the truncated enzymes with 3.5 µmol mg protein^−1^ while for Del-2, Del-3 and Del-4 are significantly higher (p<0.05), GSH-affinities reaching 8.1, 8.9 and 10.4 µmol mg protein^−1^ were measured, respectively.

### *U. unicinctus* SDO double mutant characterization

**Figure 9:**
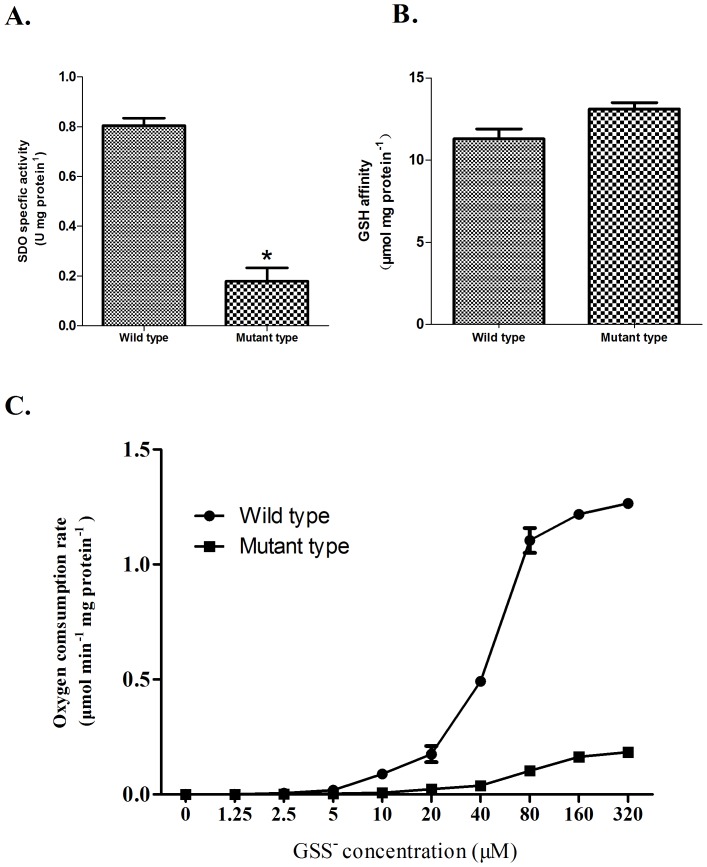
Characteristics of the *U. unicinctus* SDO mutant. A. SDO specific activities in wild type and mutant. * indicates a significant difference from the wild type (p<0.05). B. GSH affinities in wild type and mutant. C. Oxygen consumption rate versus GSS^−^ concentration in wild type and mutant.

The SDO-specific activity almost double in Del-4 compared with Del-3 when the second metal binding site became intact (Figure 8A). Based on this, D117 and H118 located in the second metal binding site (belonging to the metallo-β-lactamase superfamily signature motif HXHX**DH** (X for an arbitrary amino acid)) were chosen to be replaced by Glu and Ala, respectively, by double mutagenesis. The analysis of the double mutant D117E/H118A indicates that its SDO specific activity (0.17 U mg protein^−1^) is significantly decreased compared with wild type (Figure 9A). The reaction rates of wild type and double mutant at different substrate concentrations are shown in Figure 9C. The rates of oxygen consumption are much lower for the mutant than for wild type; a K_M_ for GSS^−^ of 174.4±16.8 µM and a V_max_ of 0.30±0.014 µmol min^−1^ mg protein^−1^ were determined for the double mutant enzyme. In contrast, the GSH affinity (13.1 µmol mg protein^−1^) is similar to wild SDO (p>0.05) (Figure 9B), indicating that the loss of SDO activity reflects the replacement of the Asp residues by Glu and His residues by Ala in the double mutant enzyme.

## Discussion

### SDO, a novel member of the metallo-β-lactamase superfamily

The members of the metallo-β-lactamase superfamily can catalyze a diversity of reactions, and are divided into 17 groups based on their biological functions [27], [28]. Glyoxalase II (GLX2) belongs to group 2 and can hydrolyze s-D-lactoylglutathione (SLG) into D-lactate and GSH [27], [29]. In this study, the *U. unicinctus* SDO protein was shown to be highly conserved among known ETHE1 (SDO) proteins, containing the signature motif HXHXDH of the metallo-β-lactamase superfamily and sharing 61% sequence identity to the *Ixodes scapularis* glyoxalase (XP_002399673.1). Because of its high similarity with GLX2, SDO is believed to be a member of group 2; for example, SDO (ETHE1) from *A. thaliana* is thought to be the putative glyoxalase II isozyme GLX2-3 [30], [31]. However, it is known that SDO cannot hydrolyze any glutathione thioesters [32], [33], [34] because it lacks several highly conserved residues (N179, Y145, F182 and D253) that participate in the hydrogen bonding of SLG in GLX2. Therefore, SDO (ETHE1) protein is suggested to belong to a new class in the metallo-β-lactamase superfamily playing an important role in GSS^−^ oxidation [13]. In our study, *U. unicinctus* SDO also lacked the GLX2 conserved amino acids (mentioned above) and instead uses oxygen to oxidize the GSS^−^ to sulfite (Table 2). Most members of the metallo-β-lactamase superfamily only display hydrolase activity, such as β-lactamases [35] and glyoxalases II [36], with the exception of the group 3 member, ROO (flavoproteins and rubredoxin oxygen: oxidoreductase) that contains two domains: a metallo-β-lactamase and a flavodoxin-like which act together to provide the oxidoreductase activity [37]. In this study, the *U. unicinctus* SDO shows oxidoreductase activity, although it only contains a metallo-β-lactamase domain. This result further supports that SDO is a novel member of metallo-β-lactamase superfamily.

As shown by the western blot, the SDO of *U. unicinctus* is located in the mitochondria (Figure 4), which was in accordance with previous reports of human and *A. thaliana* ETHE1s [14], [34]. Interestingly, it seems that separation of the *SDO* gene and *rhodanese* gene occurred during the mitochondrial occurrence in eukaryotes during the evolution of the species. Indeed in some bacteria, such as *Methylobacter tundripaludum*, *Nitrosococcus watsonii* and *γ-proteobacterium*, a SDO-like amino acid sequence is linked with a rhodanese-like domain (Figure 6A). However, in eukaryotes, the *SDO* gene and *rhodanese* gene are assigned as two separate genes in the NCBI database. In addition, we found that the SDO-like amino acid sequence in *γ-proteobacterium* was clustered with that of eukaryotes (Figure 6B). Considering that early eukaryote mitochondria are thought to be derived from intracellular bacterial symbionts of proteobacterial origin [38] it is therefore suggested that the *SDO* gene and *rhodanese* gene were separated when the endosymbiont genes were integrated into the eukaryote genomes.

### SDO catalytic activity

Hildebrandt and Grieshaber [10] reported maximal rates for SDO purified from rat liver and lugworm body-wall tissue of 0.87±0.04 and 0.85±0.24 U mg protein^−1^, respectively, similar to 0.80 U mg protein^−1^ measured for *U. unicinctus* SDO conversion of GSS^−^ to sulfite. However, a significantly lower K_M_ (82.5±9.9 µM) was determined in this latter enzyme as compared to human (340±30 µM [15]) or thiobacilli (120–240 µM [39]) SODs, indicating that SDO from *U. unicinctus* binds the substrate tighter than those from other animals.

The members of the metallo-β-lactamase superfamily usually possess two potential metal binding sites [28], which in *U. unicinctus* SDO were predicted to be formed by the residues H113, H115, H169 and D188 for metal I binding site and D117, H118, H169 and H229 for metal II binding site. We show that the two metal binding sites are important for enzyme activity: the activity decreased steeply from the complete form to the increasingly truncated forms of the protein (Figure 8A). For example, the activity increased 2.8-fold and 5.1-fold in Del-3 (completed metal I binding site) and Del-4 (completed both metal binding sites) respectively as compared to Del-1 (no metal binding site). No significant differences (*p*>0.05) were detected for the K_M_ among the Del-2, Del-3, Del-4 or wild type enzyme (Table 2). Bugg [40] suggested that in the dioxygenase superfamily, the metal iron center is important for substrate binding and activation of the catalytic reaction. Therefore, this may explain the almost invariable affinity for the substrate GSS^−^ in the truncated proteins. In this study, the K_M_ decreased more obviously from Del-3 to Del-2 than from Del-4 to Del-3. Because only one metal ion is found in SDO, these results suggest that the metal ion is most likely located in the metal I binding site, a suggestion in agreement with a recent report where only one Fe^2+^ was found in the metal I binding site in *A. thaliana* [34]. McCoy et al. [31] reported that the metal II binding site usually directly coordinate to iron in other metallo-β-lactamase group members with only one metal iron. In our study, the metal II binding site integrity may also affect the SDO catalytic activity as a 388 µM^−1^ s^−1^ increase in catalytic efficiency (k_cat_/K_M_) was observed indicating the metal II binding site is important for the enzyme activity. Moreover, the double mutant D117E/H118A, in the metal II binding region, shows approximately one-fifth of the wild type specific activity. The K_M_ of the double mutant is 174.4±16.8 µM, two fold higher than that of the wild type, indicating also a reduced affinity for binding the substrate. In many metallo-β-lactamases, substitution of the homologous Asp and His can impair the enzyme activity, as Asp coordinates the metal ion for correct substrate binding [41]. In addition, dissociation of H^−^ from OH_2_ binding of the metal ion is suppressed in the metallo-β-lactamase (IMP-1) mutant D120E [42]. However, dissociation of H^−^is the most important step for the binding of the substrate GSS^−^ [15]. Taken together, these are thought to be the major reasons for the increase in the K_M_ and the decrease in V_max_ in the double mutant D117E/H118A.

The catalytic efficiency (k_cat_/K_M_) is 822.00 µM^−1^ s^−1^ and 1327.22 µM^−1^ s^−1^ for Del-4 and wild type enzyme, respectively. This difference can be attributed to differences in the number of GSH binding sites [15]. The predicted amino acid for GSH binding in *U. unicinctus* SDO are Arg197, Tyr231, Met279 and Ile283 according to its sequence homology with human GLX2. In human GLX2, the backbone amino group of Lys143 and the side chain of Tyr175 were shown to bind the cysteine portion of GSH via hydrogen bonds while the side chains of Arg249 and Lys252 establish hydrogen bonds with the glycine portion of GSH but overall [32]. In this study, Del-1 and Del-2 contained no GSH binding site; Del-3 contained Arg197, and Del-4 contained Arg197 and Tyr231. The increase in GSH affinity for Del-4 compared with Del-3 indicated that Tyr231 in *U. unicinctus* SDO (Figure 8B) is homologous with Tyr175 in human GLX2 the key residue for GSH binding. However, the decreased GSH binding ability of other amino acids such as Arg197, Met279 and Ile283 as compared with that of Tyr231 (Del-3), may be caused by mutations in the homologous human amino acids. In addition the reason behind the significant increase in GSH binding affinity for Del-1 to Del-2 may result from the fact that the metal also combines with GSH because of its similarity in structure to GSS^−^. The binding to GSH could compete with the binding of GSS^−^, but may be helpful to promote the oxidation of GSS^−^. In humans, the K_m_ for GSS^−^ of SDO is slightly lower in the presence of GSH with a higher SDO specific activity [15]. Although the Del-1 construct did not contain the predicted GSH binding site, its GSH binding affinity is still as low as 3.5 µmol mg protein^−1^. We suppose that an unknown GSH binding region exists in the Del-1 construct, which non-enzymatically enhances GSS^−^ oxidation with an overall increase in SDO specific activity. However, further research into the nature of the GSH binding sites is required.
